# Pre-endodontic restoration of structurally compromised teeth: current concepts

**DOI:** 10.1038/s41415-021-3467-0

**Published:** 2021-09-24

**Authors:** Dimitrios Gavriil, Angeliki Kakka, Paul Myers, Christopher J. O´Connor

**Affiliations:** 1 MClinDent in Restorative Dentistry, Department of Restorative Dentistry, School of Dental Sciences and Dental Hospital, Newcastle University, Framlington Place, Newcastle upon Tyne, UK; 2 General Dental Practitioner, Private Practice, Athens, Greece; 3 Speciality Doctor in Restorative Dentistry, Department of Restorative Dentistry, School of Dental Sciences and Dental Hospital, Newcastle University, Framlington Place, Newcastle upon Tyne, UK; 4 Clinical Fellow in Restorative Dentistry, Department of Restorative Dentistry, School of Dental Sciences and Dental Hospital, Newcastle University, Framlington Place, Newcastle upon Tyne, UK

## Abstract

Teeth that require endodontic treatment are often structurally compromised and this considerably complicates endodontic procedures. Therefore, pre-endodontic restoration is a key approach that dentists should consider for such teeth. This article discusses current concepts of pre-endodontic restoration, with a focus on adhesive restorative methods and surgical/orthodontic techniques, and provides a relevant decision-making flowchart.

## Key points

- Highlights the importance of pre-endodontic restoration for the predictability of endodontic treatment.
- Discusses restorability aspects for structurally compromised teeth.
- Reviews current restorative and surgical/orthodontic techniques for pre-endodontic restoration and provides a decision-making flowchart.

## Introduction

The purpose of endodontic treatment is to prevent or treat apical periodontitis by maintaining an aseptic root canal system or disinfecting this when previously infected.^1^ Rubber dam isolation of the subject tooth is considered mandatory as it prevents ingress of oral bacteria and saliva, precludes inhalation and ingestion of instruments, and averts leaking of irrigation solutions into the oral cavity.^1^

Teeth that require endodontic intervention are often structurally compromised due to conditions such as caries, trauma, or root resorption. The limited residual tooth tissue substantially complicates endodontic procedures. Considering pre-endodontic restoration before initiating endodontic treatment is valuable for compromised teeth as this approach:

- Simplifies optimal rubber dam isolation for the subsequent endodontic visits^2^
- Creates space for prolonged function of irrigation solutions^3^
- Allows for effective inter-appointment temporisation to prevent bacterial microleakage, seepage of intracanal medicaments and gingival ingrowth into the cavity^4^
- Prevents fractures of the weakened tooth structure, thus maintaining repeatable reference points^5^
- Improves aesthetics during the endodontic treatment period, thus enhancing patients' acceptance
- Facilitates post-endodontic restoration.

While traditional non-adhesive techniques of pre-endodontic restoration, such as amalgam core build-up, copper bands or temporary crowns, may still prove useful for some clinicians when appropriately performed,^6^ they also present with many shortcomings which, along with the development of adhesive approaches, have limited their clinical value for this purpose.^7^^,^^8^ These techniques will not be further considered in this article but the reader may refer to Table 1 for a brief overview.

**Table 1:** Overview of traditional non-adhesive techniques of pre-endodontic restoration

Technique	Advantages	Disadvantages
Amalgam core build-up	Durable with long track record for restoring badly broken down teeth May be useful for clinical situations where bonding is unpredictable	Amalgam is being phased out Mechanical retention is required; this often necessitates additional tooth tissue removal or use of dentine pins Endodontic treatment needs to be delayed due to the setting properties of amalgam Amalgam particles may block root canal system Aesthetics
Copper/orthodontic bands with temporary cements	May increase fracture resistance of compromised teeth	Periodontal complications due to poor marginal adaptation and impaired oral hygiene Suboptimal contours and occlusion Risk of dislodgement Root canal blockage by cement particles Aesthetics and patient comfort
Temporary crown	Restoration of occlusion and aesthetics	Sufficient residual tooth tissue required to retain a crown Risk of dislodgement Compromised visibility Potential patency impairment by cement particles

The aim of this article is to provide an evidence-based overview of modern concepts of pre-endodontic restoration focusing on restorative techniques with bondable materials and surgical/orthodontic techniques that expose tooth tissue.

## Restorability considerations

Restorability assessment is essential before embarking on endodontic treatment. This should include evaluation of the restorative status of the tooth (structural, periodontal, endodontic), as will be discussed in the following sections, although local and general factors relating to the context of treatment (for example, tooth used as an abutment for fixed or removable prostheses, parafunction, medical history, patient expectations, cost) should also be examined.^9^

Certain restorability indices^9^^,^^10^ have been developed to aid decision-making by quantifying clinical judgements; however, their validity is not sufficiently tested and subjective elements are still included.

### Structural assessment

Clinicians should be cognisant that typical clinical and radiographic examination may have limited sensitivity in detection of caries, cracks and marginal deficiencies.^11^ Therefore, definitive assessment of restorability should be performed only after total removal of previous restorations, caries and unsupported tooth tissue.

The importance of a circumferential 1.5-2 mm band of healthy tooth tissue, namely ferrule effect, is well established for a positive prognosis, yet current evidence suggests that presence of a partial ferrule (1-3 walls) may be sufficient.^12^

Other biomechanical parameters, such as residual cusp thickness and loss of marginal ridges, also need to be assessed as these have been shown to impact on tooth stiffness.^13^ Besides, fracture resistance can be enhanced by preserving pericervical dentine through conservative access cavity preparation.^14^

### Periodontal considerations

Another consideration is the potential infringement of deeply subgingival restoration margins within the supracrestal tissue attachment (STA),^15^ formerly known as biologic width, which consists of the junctional epithelium and supracrestal connective tissue attachment. STA violation is believed to trigger adverse periodontal effects, although the specific aetiology (biofilm, trauma, material toxicity, combination of factors) is not clear.^15^ Mean STA apico-coronal dimension is found to be 2.15-2.30 mm; however, considerable variability exists according to tooth type, site, periodontal health and healing time after previous surgery.^16^ Thus it would be prudent not to rely on mean values but to attempt measuring the STA dimension at each individual situation. A simple method is by transgingival probing, which is considered comparably reliable to direct bone sounding after flap reflection,^17^ although it might be influenced by probing force^18^ and the inflammatory state of the tissues.^19^ Other researchers suggest accounting for the full supracrestal gingival tissue (including gingival sulcus) to address variations in sulcus depth.^20^

**Fig. 1:**
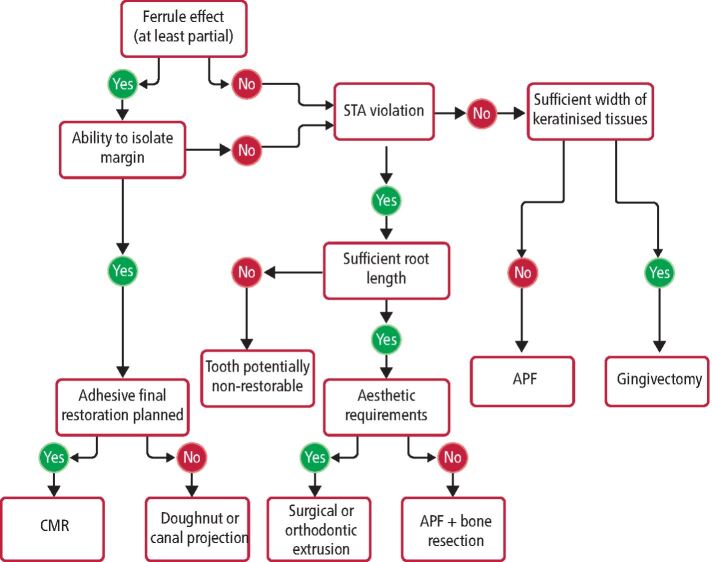
Decision-making flowchart for pre-endodontic restoration

Many other factors (Box 1), such as having a favourable crown-to-root ratio (up to 1:1)^21^ and width of keratinised tissues (≥3 mm),^22^ may influence restorability as well as the technique selection for pre-endodontic restoration (Fig. 1).

Box 1 Periodontal assessment Factors to consider:

- Periodontitis (unstable disease, amount of bone loss)
- STA violation
- Mucogingival dimensions (width/thickness of keratinised tissues, recession)
- Crown-to-root ratio
- Furcation defects
- Mobility (grade, progressive mobility, occlusal trauma)
- Root morphology (grooves, concavities, root proximity).

### Endodontic considerations

Clinicians should also assess whether the subject tooth is amenable to endodontic treatment (primary, secondary or surgical). This requires evaluation of several parameters, such as the presence and extent of periapical pathology, root damage (fractures, resorption, perforation), complexities in root canal system (obliteration, separated instruments) and proximity to adjacent anatomical structures.^9^

### Planning final restoration

Following restorability assessment, it is crucial that the definitive restoration of the tooth is decided at this early stage as this could also influence the technique selection for pre-endodontic restoration (Fig. 1). In addition, when a cuspal coverage final restoration is planned, cusp reduction may be performed at pre-endodontic stage to improve visibility and protect the tooth from fractures.^5^

## Restorative techniques

According to the above, a pre-endodontic restorative technique is indicated for teeth with at least partial ferrule as well as cavity margins that enable moisture control and do not infringe into the STA (Fig. 1). A summary of current methods, using materials that can bond to tooth tissue, is provided in the following sections.

### Cervical margin relocation

Relocating a deep cervical margin to a supragingival position using resin composite was first described by Dietschi and Spreafico^23^ as 'cervical margin relocation' (CMR). Other researchers have termed this technique as 'deep margin elevation'^24^ or 'proximal box elevation'.^25^

**Fig. 2:**
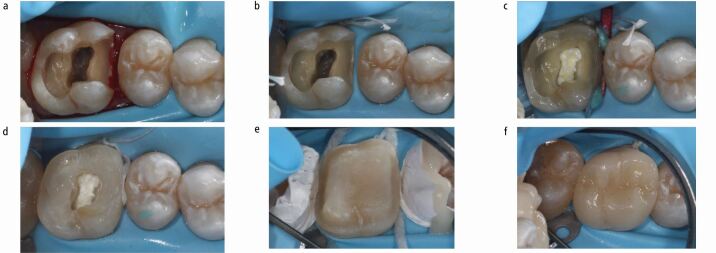
Cervical margin relocation (CMR): from pre-endodontic to post-endodontic restoration. a) Non-vital upper right first molar after removal of caries and previous restorations. b) Isolation and c) mesial CMR with sectional matrix. d) Pre-endodontic restoration (including cusp reduction) completed. e) Preparation for adhesive restoration after completion of endodontic treatment. f) Ceramic onlay after adhesive cementation

This approach is highly indicated when an adhesive final restoration is planned;^24^ for example, in case of a localised deep margin where the remaining walls provide sufficient enamel for bonding (Fig. 2). More specifically, it allows for immediate dentine sealing (IDS) to be performed in freshly cut dentine just before relocating the margin and this prevents dentine contamination, enables bond maturation and enhances bond strength of the subsequent indirect restoration.^26^ A second IDS can be performed after completion of endodontic treatment to provide an immediate seal of the root canal obturation and optimise the cavity for the indirect restoration by blocking undercuts.^24^ Moreover, the supragingival location of the margin aids the subsequent procedures of impression taking and final cementation.^24^

The main limitation of CMR is that adequate seal of the margin with a well-fitted matrix under rubber dam isolation is considered as a necessary prerequisite,^24^ although this is not always achievable, especially for subgingival margins. In addition, sufficient coronal walls should be remaining in order to support the matrix,^24^ which can be reduced at thin sections so as to be able to slide subgingivally and provide a tight seal. Stabilisation of the matrix can be achieved by packing of polytetrafluoroethylene (PTFE) tape (as wedging is usually not feasible) or by the double matrix technique (a sectional matrix inside a circumferential matrix) in deep localised cavities.^24^

CMR has been described with either packable or flowable composite, which have shown comparable marginal integrity *in vitro*.^27^ Marginal adaptation can be optimised by placement of packable composite in small increments rather than a single layer,^25^ decreasing its viscosity through pre-heating^28^ and limiting the overall thickness of flowable composite to 1-1.5 mm due to its higher shrinkage and lower filler content.^29^

### Doughnut technique

**Fig. 3:**
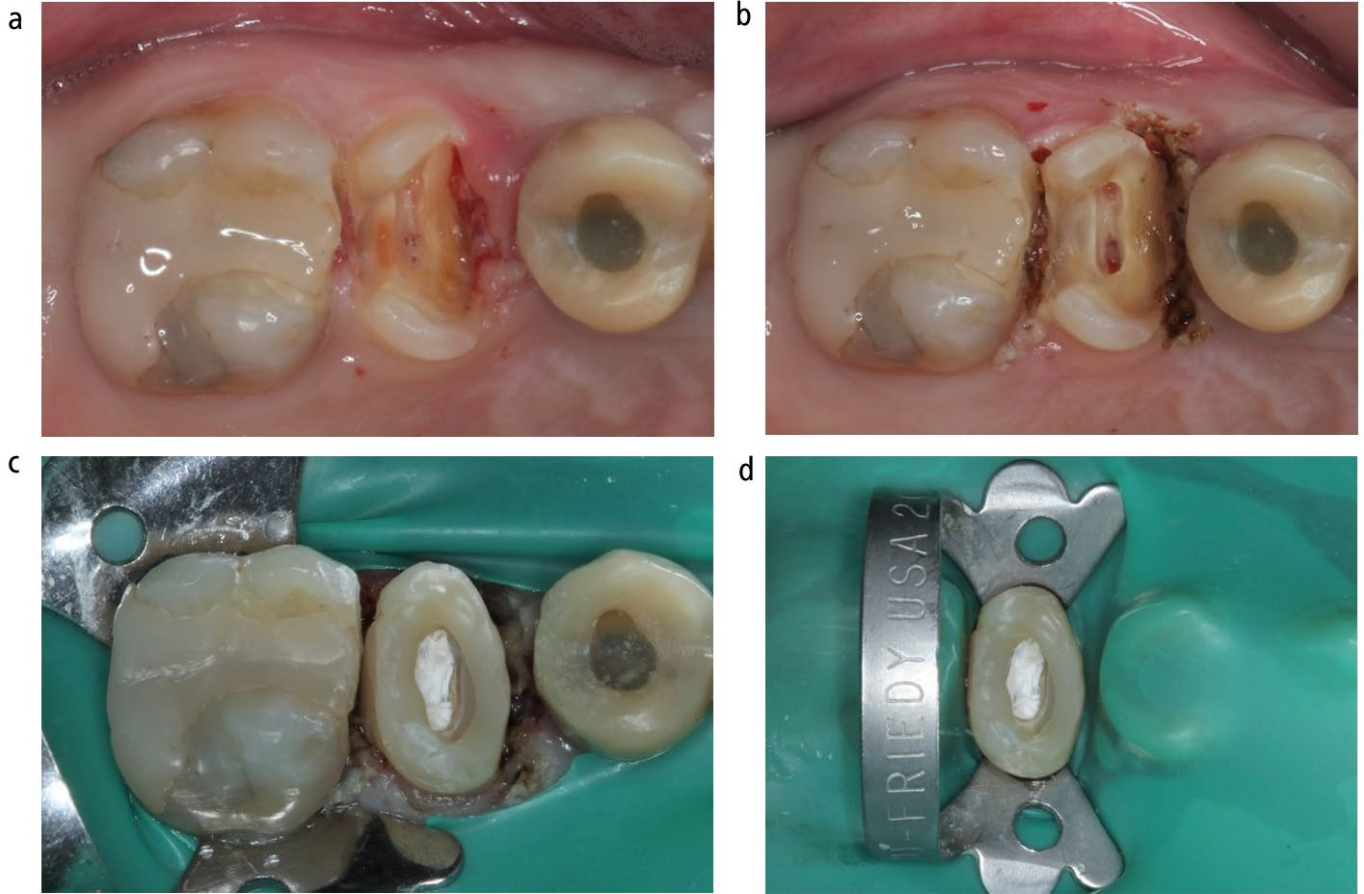
Combined pre-endodontic restoration of an upper right second premolar. a) Pre-operative condition with subgingival mesial and distal margins. b) Following laser gingivectomy. c) 'Doughnut' composite build-up using polytetrafluoroethylene in pulp chamber. d) Final rubber dam isolation before initiating endodontic treatment

Different approaches may be considered when a non-adhesive final restoration is planned (for example, conventional crown); for example, in teeth with multiple missing walls and a large amount of peripheral margin into dentine. In these situations, matrix stability is usually not achievable,^24^ and free-hand^30^ or two-step (free-hand followed by secondary placement of matrix)^31^ pre-endodontic core build-ups may be applied as an interim solution to facilitate endodontic treatment before definitive crown preparation (Fig. 3).

The so-called 'doughnut' technique involves a circumferential build-up of the cavity walls and use of a suitable barrier (cotton pellet, thermoplastic gutta-percha, PTFE, liquid dam) to prevent blockage of root canal orifices (Fig. 3c). This method is mainly described with free-hand flowable composite build-up after using retraction cord in the sulcus to displace the soft tissues.^3^^,^^30^

Advantages include the relatively simple application and the maintenance of access to the root canal system that prevents complications in canal location and patency.^30^ Concerns arise regarding marginal adaptation and management of overhangs, especially when applied free-hand, although it could be argued that the cervical proportion of the material will be removed during the subsequent crown preparation to natural tooth tissue margins.

Following completion of endodontic treatment, and before proceeding to the final composite build-up of the 'doughnut' cavity, clinicians may consider fibre reinforcement of the peripheral walls to enhance the stress-absorbing capacity of the core.^32^

### Canal projection

As an alternative to the above technique, this approach involves core build-up with projection of root canal orifices from the pulp chamber floor to the cavosurface.^33^ Canal projection provides better visualisation and straight-line access to the canals, canal individualisation in case of close proximity of canal orifices on the chamber floor, correction of misdirected access cavity and enhanced hydraulic condensation of obturation materials as well as adequate sealing and reinforcement of the chamber floor or perforation repair materials.^2^^,^^33^

**Fig. 4:**
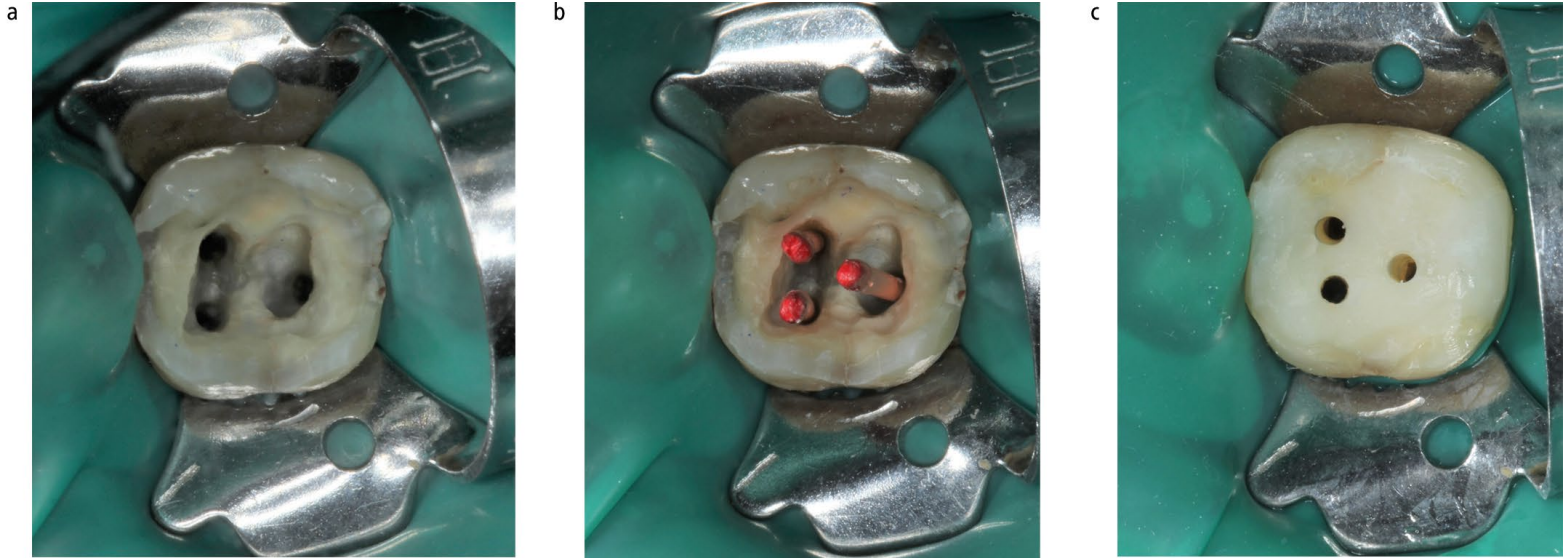
Canal projection on a lower left second molar. a) Access (after removing caries/previous restorations) and flaring of coronal third of root canals. b) Greater taper gutta-percha points to preserve canal patency. c) Finalised pre-endodontic composite build-up with projected root canals to the cavosurface

This technique has been described with the dedicated Projector Endodontic Instrument Guidance System (PEIGS; CJM Engineering, USA), which consists of a tapered plastic device sliding onto an endodontic instrument to preserve canal patency.^33^ Alternative methods, including use of hypodermic needles^34^ or Greater Taper gutta-percha points (Fig. 4),^2^^,^^3^ are easily accessible, more affordable and offer equivalent results.^2^^,^^3^

A relevant drawback compared to the 'doughnut' might be the more time-consuming procedure for the build-up as well as for the temporisation between endodontic visits, as each projected canal needs to be temporised as a separate cavity.

A modification of this method can also be applied for pre-endodontic restoration of cervical lesions with pulp involvement, such as extensive Class V cavities^3^ or external cervical resorption,^35^ in order to maintain patency to the root canal system.

### Open-sandwich technique

This technique is mainly described with an intermediate layer of resin-modified glass-ionomer cement (RMGIC) as a base for overlying composite build-up.^36^ It has been advocated due to the advantages of using glass-ionomer in deep margins compared to composite, such as less dependence on moisture control,^37^ caries inhibition due to fluoride release^38^ and inherent affinity to dentine, although with lower bond strength.^39^

On the other hand, whether RMGIC prevents microleakage at its interfaces with dentine^40^ and composite^41^ is questionable. In addition, it appears to be subjected to water sorption and crack formation that may extend to the underlying dentine.^42^ RMGIC is also less suitable when calcium silicate cements are used in the pulp chamber (for example, perforation repairs or vital pulp therapy) as it achieves a weaker bond to these materials compared to composite.^43^

Pure calcium silicate cements, such as Biodentine (Septodont, France), have also been used for the open-sandwich technique in combination with overlying composite build-up, especially in vital pulp therapy cases.^44^ However, more long-term data would be necessary due to their low mechanical properties and bond to dentine that is considerably inferior to composite and requires a seven-day maturation to become at least comparable to glass-ionomer.^45^

Taking the above into consideration and despite their decreased technique-sensitivity, open-sandwich approaches do not appear to offer any significant benefit over composite techniques for pre-endodontic restoration.

### Periodontal implications

A common concern regarding restorative techniques is the potential impact on periodontal tissues. When not impinging on STA, well-fitted subgingival composite restorations can conform with periodontal health and clinical attachment gain in patients with healthy periodontium and stringent oral hygiene.^46^ When infringing within the STA, composite CMR is reported to induce higher bleeding on probing after 12 months;^47^ however, when provided under flap (to eliminate the technique-sensitivity of producing a well-fitted restoration at deeply subgingival margins), it appears to result in comparable six-month tissue response to supragingival restorations placed after surgical crown lengthening (SCL).^48^ Nevertheless, more long-term data would be needed as degradation of CMR composite may take place over time, especially after three years.^49^ Also, placing subgingival restorations under flap still requires a surgical procedure not much different from that of conventional crown lengthening along with its potential drawbacks such as gingival recession.^50^

## Surgical/orthodontic techniques

Restorative techniques may be limited in certain clinical situations (Fig. 1), especially when:

- Ferrule effect needs to be enhanced
- STA violation is expected
- Isolation of the cavity is not possible (for example, gingival ingrowth into cavity).

Techniques that can expose tooth tissue may be employed in such cases, either alone or in combination with a restorative build-up (Fig. 3).

### Surgical crown lengthening

This approach involves either an apically positioned flap (APF) (with or without osseous resection) or a flapless gingivectomy procedure aiming to expose more tooth tissue in structurally compromised teeth.

Gingivectomy may generally result in less post-operative morbidity than flap surgery,^51^ and is indicated when there is sufficient width of keratinised tissues (≥3 mm)^22^ and no STA violation is expected.^52^ A suitable example would be a case of altered passive eruption, where gingival margin is coronally placed over enamel.^53^ Techniques to perform gingivectomy include scalpel, electrosurgery and lasers (Nd:YAG, CO_2_, diode). Scalpel is considered as the 'standard treatment', and although more cost-effective, it is often associated with peri-operative haemorrhage;^51^ thus, it may complicate isolation for the endodontic treatment to be performed at the same visit. In comparison with scalpel technique, electrosurgery is advantageous in terms of haemostasis, incisional time and post-operative discomfort,^54^ although when inappropriately performed, it may result in delayed healing and tissue necrosis.^55^ Lasers have comparable advantages with electrosurgery regarding bleeding control, ease of use and post-operative pain,^56^ and are generally superior to scalpel in terms of incision accuracy, sterilisation of the surgical field, patient tolerance, tissue rebound and scarring,^51^^,^^57^ but they are costly and might cause collateral thermal damage.^51^

APF is indicated when there is insufficient width of keratinised tissues (<3 mm)^22^ and/or osseous resection is required to re-establish STA apico-coronal dimension.^52^ This approach may be necessary in altered active eruption cases, where cementoenamel junction coincides with crestal level.^53^ Nevertheless, significant tissue rebound is expected, especially within 3-6 months post-surgery,^58^^,^^59^ and appears to be associated with several factors, such as thick gingival phenotype, amount of bone removal and flap positioning at crestal level.^58^ The extended healing time could delay the provision of definitive restoration, jeopardising the outcome of endodontic treatment.^60^ SCL with bone removal may also result in more than a twofold risk of extraction of endodontically treated posterior teeth after ten years,^61^ potentially due to the negative impact on crown-to-root ratio^61^ and furcation exposure.^62^ Long-term clinical studies indicate that approximately half of endodontically treated teeth with osseous resective SCL will be lost after 10-13 years.^61^^,^^63^

In addition, as bone removal often extends to a wider area to prevent disharmony in soft tissue contours, it affects adjacent and non-adjacent sites, causing long clinical crowns, black triangles and papillae loss.^50^ Consequently, SCL with bone resection is usually not recommended in the aesthetic zone and alternatives, such as orthodontic or surgical extrusion, may be considered instead.

### Orthodontic extrusion

Orthodontic extrusion is often preferable to SCL, principally in the aesthetic zone, as it is more conservative, averts bone resection, and secures the root contours and periodontal integrity of the treated and adjacent teeth,^50^^,^^64^ while it is highly indicated in patients whose medical condition precludes surgical approaches.^65^

This technique induces coronal migration of the supporting bone and soft tissues, especially when performed under low-intensity forces (slow extrusion), which may be desirable in certain cases (angular bony defects, lack of keratinised tissues),^66^ but may also cause aesthetic problems that require secondary surgical correction.^67^ Nevertheless, this can be avoided with combined supracrestal fibrotomy during the extrusion period.^68^ Rapid extrusion (with strong traction forces) results in less coronal displacement of periodontal tissues,^69^ but requires an extended retention period and is associated with a higher occurrence of ankylosis^67^ and root resorption.^70^

Orthodontic extrusion is generally contraindicated in cases of short roots, ankylosis, hypercementosis, furcation exposure, root proximity and premature closure of embrasures.^71^ Further limitations of this method include long treatment duration, patient compliance, unfavourable aesthetics, high cost, availability of proper anchorage, impairment of oral hygiene and risk of relapse.^71^^,^^72^

### Surgical extrusion

Surgical extrusion involves the intentional coronal displacement of the remaining tooth structure within the socket, with or without rotation. Rotation may be employed in order to shift subgingival fracture lines to a more favourable position, limiting the amount of extrusion required, as well as to increase cervical width and avoid unwanted black triangles due to the narrower root diameter of extruded teeth.^73^^,^^74^

Unlike orthodontic extrusion, this technique is typically not applicable to molars^75^ but is reported to produce comparable results with regards to periodontal healing,^76^ marginal bone loss and root resorption.^77^ It also consists of a simple one-step procedure, which encourages patients' cooperation and minimises treatment time and cost,^73^^,^^74^ as well as maintaining gingival architecture.^78^ Additionally, no cases of ankylosis concerning surgically extruded teeth have been described in the literature^73^^,^^74^^,^^78^^,^^79^ and short-term non-rigid splinting may be an additional preventive measure.^74^

Contraindications include anatomical variations (such as hypercementosis), inadequate root length and divergent or thin roots.^75^^,^^79^ Surface (non-progressive) root resorption is the most frequent complication (30%) while progressive root resorption is rare (3.3%) and it had mostly been associated with earlier flap approaches which included luxation forces to the apex.^74^ Less traumatic techniques using periotomes^78^ or a vertical extraction system^79^ limit the risk of resorption, although the former may still transfer lateral forces to the socket,^75^ while the latter may risk perforation as it works via a self-tapping screw anchored into the root canal.^79^ Other complications of surgical extrusion include slight mobility and marginal bone loss, which both seem to be more associated with premolars,^79^^,^^80^ while the incidence of tooth loss is low (5%).^74^

In the literature, it is often suggested to fill root canals of teeth during surgical or orthodontic extrusion with calcium hydroxide^71^^,^^74^^,^^78^ in order to prevent resorption activity due to its antibacterial and healing properties. This might be applicable in cases where initiation of endodontic treatment is urgent; however, concerns arise regarding the ability to achieve adequate isolation.^81^ Endodontic treatment can be completed after splint removal or during the healing phase as long as splinting type and location allow proper access and placement of the rubber dam.^75^

## Conclusion

It is evident that pre-endodontic restoration has many advantages for the predictability of endodontic treatment for structurally compromised teeth and clinicians have a plethora of techniques to employ. Considering that high-quality evidence in this field is still limited, future controlled studies comparing the effect of available methods on the outcomes of endodontic treatment would be highly recommended.
